# Structural Details of Ufd1 Binding to p97 and Their Functional Implications in ER-Associated Degradation

**DOI:** 10.1371/journal.pone.0163394

**Published:** 2016-09-29

**Authors:** Le Thi My Le, Wonchull Kang, Ji-Yun Kim, Oanh Thi Tu Le, Sang Yoon Lee, Jin Kuk Yang

**Affiliations:** 1 Department of Chemistry, College of Natural Sciences, Soongsil University, Seoul 156–743, Korea; 2 Department of Biomedical Sciences and Chronic Inflammatory Disease Research Center, Ajou University School of Medicine, Suwon 443–721, Korea; 3 Department of Information Communication, Materials, and Chemistry Convergence Technology, Soongsil University, Seoul 156–743, Korea; University of Pittsburgh, UNITED STATES

## Abstract

The hexameric ATPase p97 has been implicated in diverse cellular processes through interactions with many different adaptor proteins at its N-terminal domain. Among these, the Ufd1-Npl4 heterodimer is a major adaptor, and the p97-Ufd1-Npl4 complex plays an essential role in endoplasmic reticulum-associated degradation (ERAD), acting as a segregase that translocates the ubiquitinated client protein from the ER membrane into the cytosol for proteasomal degradation. We determined the crystal structure of the complex of the N-terminal domain of p97 and the SHP box of Ufd1 at a resolution of 1.55 Å. The 11-residue-long SHP box of Ufd1 binds at the far-most side of the Nc lobe of the p97 N domain primarily through hydrophobic interactions, such that F225, F228, N233 and L235 of the SHP box contact hydrophobic residues on the surface of the p97 Nc lobe. Mutating these key interface residues abolished the interactions in two different binding experiments, isothermal titration calorimetry and co-immunoprecipitation. Furthermore, cycloheximide chase assays showed that these same mutations caused accumulation of tyrosinase-C89R, a well-known ERAD substrate, thus implying decreased rate of protein degradation due to their defects in ERAD function. Together, these results provide structural and biochemical insights into the interaction between p97 N domain and Ufd1 SHP box.

## Introduction

p97, also known as VCP (valosin-containing protein), is a hexameric ATPase of type II AAA+ family [1]. Each p97 protomer comprises an N-terminal domain (hereafter N domain), two ATPase domains in tandem (D1 and D2) which form doubly packed hexameric rings, and a short C-terminal tail region [2–5]. p97 has been implicated in a variety of cellular processes, such as ER-associated degradation, post-mitotic Golgi reassembly, cell cycle regulation, apoptosis, DNA damage response, mitochondria quality control, and autophagy [6]. p97 is among the most abundant cellular proteins, representing ~1% of total cellular protein [7], which may reflect its diverse cellular functions. In contrast to this diversity, the molecular activity of p97 itself is rather simple, involving the hydrolysis of ATP to generate large conformational changes [5,8]. Thus, the functional diversity of p97 is realized through interactions with many different adaptor proteins, which are primarily recruited to the N-terminal domain, although some adaptors also bind the C-terminal tail [9,10].

Among the large number of adaptor proteins, the most well studied are two major adaptors: p47 and the Ufd1-Npl4 heterodimer. p47 has been implicated in cellular processes accompanying membrane fusion events, such as post-mitotic Golgi reassembly [11], whereas the Ufd1-Npl4 heterodimer was initially characterized in ER-associated degradation [12,13]. However, a growing number of studies have reported that p97-Ufd1-Npl4 complex is involved in the proteasomal degradation of various proteins, not only from the ER membrane but also from other cellular locations, such as the nucleus, mitochondrial membrane, ribosome and even cytosolic multi-protein assemblies [14–18]. Thus p97 inhibitors are under development as anti-cancer therapeutic agents, which target the proteasomal stress of cancer cells in a similar manner to proteasome inhibitors [19].

p97 and Ufd1-Npl4 form a complex with a stoichiometry of 6:1 [20], such that one elongated bilobed Ufd1-Npl4 heterodimer sits on the peripheral region of the p97 hexamer ring, as demonstrated via electron microscopy analyses [20,21]. Then the p97-Ufd1-Npl4 complex extracts ubiquitinated client proteins from their holding environment, subcellular membrane or multi-protein complex, for subsequent proteasomal degradation [22].

**Fig 1:**
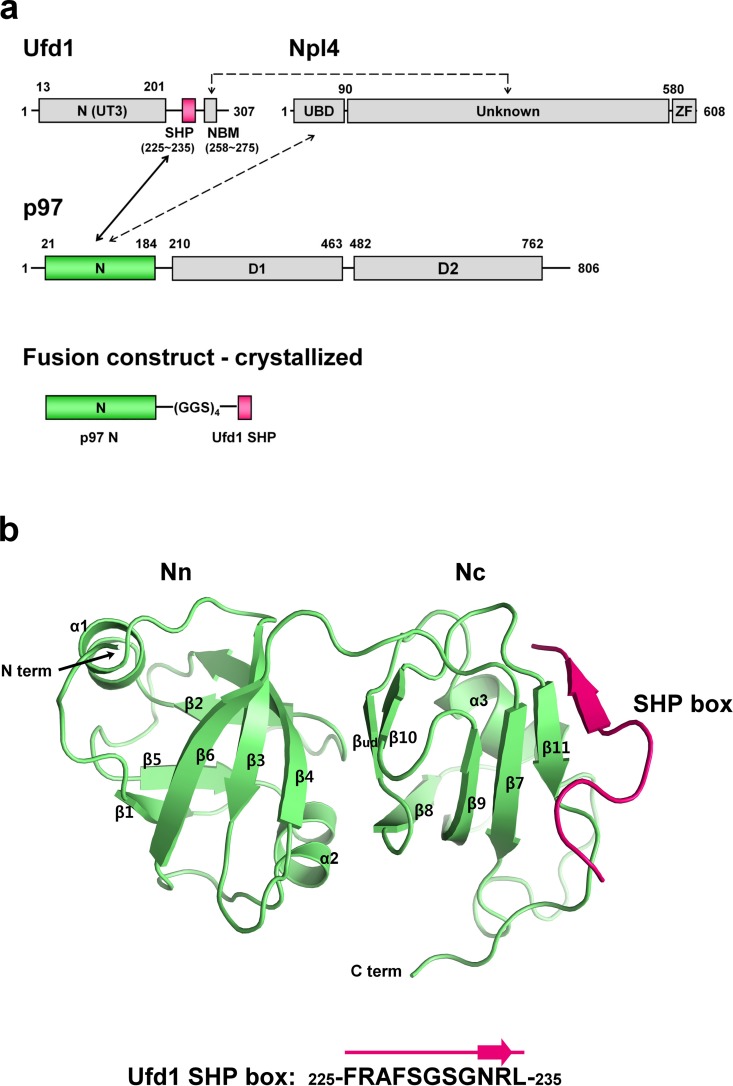
Structure of the complex between p97 N domain and Ufd1 SHP box. **(a)** *Upper*—domain composition of Ufd1, Npl4 and p97 and their interaction profile; *lower*—design of the fusion construct for the crystal structure determination. The Ufd1 SHP box and p97 N domain, examined in this study, are colored in pink and green, respectively, and the other regions are in gray. The NBM region in Ufd1 denotes an Npl4 binding motif region (258–275), previously identified by Bruderer *et al*. (2004) [23]. **(b)** Overall structure of p97 N domain (green) with a bound Ufd1 SHP box (pink). Secondary structural elements of the p97 N domain are noted in accordance with the previous report by Zhang *et al*. (2000) [3], except for β_ud_ (β-strand undefined previously) which was newly assigned in the present study.

The Ufd1-Npl4 heterodimer has two binding modules for the p97 N domain: the UBD (residues 1–80; ubiquitin-like domain; also called ULD, UBX-like, UBXL) of Npl4 and the SHP box (residues 225–235; also called BS1, binding site 1) of Ufd1 (Fig 1A) [23,24]. Similarly, p47, the other major p97 adaptor, also contains two p97N-binding modules: the UBX domain and the SHP box. Thus, the two major adaptors, Ufd1-Npl4 and p47, share a common mechanism of bipartite binding to p97, in that both adaptors contain a SHP box and that the Npl4 UBD is similar to p47 UBX in the binding site as well as in the overall fold [23,24].

To gain insights into the assembly principle and the molecular mechanism of p97-Ufd1-Npl4 segregase complex, we initiated a series of structural and biochemical studies on this complex. In this initial report, we present the crystal structure of the complex between the p97 N domain and the Ufd1 SHP box at a high resolution of 1.55 Å. Based on the complete atomic details of this interaction, we further explored the functional implications of this complex using binding experiments and cell-based ER-associated degradation assays.

## Material and Methods

### Plasmid construction

Overlap extension polymerase chain reaction (or OE-PCR) method [25] was used to generate the coding DNA’s for two different kinds of fusion polypeptide containing p97 N domain (21–196; p97N) and Ufd1 SHP box (225–235; Ufd1SHP): p97N-(GGS)_4_-Ufd1SHP and Ufd1SHP-(GGS)_4_-p97N. The coding DNA fragment was treated with *BamHI* and *XhoI*, and then ligated into pVFT3S bacterial expression vector which tags His_6_-thioredoxin to the N-terminus of a target protein [26]. The expression vectors for the mutants were produced by the method of QuikChange Site-Directed Mutagenesis Kit (Stratagene, USA). Mammalian expression vectors of the wild type and mutants of p97 and Ufd1 are constructed based on pcDNA3-HA (*XbaI-ApaI* sites) and p3XFLAG-CMV-10 (*HindIII-XbaI*), respectively. The plasmid of N-terminal Myc-tagged tyrosinase mutant (C89R) cloned into pcDNA3.1 vector was provided by Petek Ballar (Ege University, Turkey).

### Preparation of protein and peptide samples

The fusion proteins were over-expressed in *E*. *coli* strain Rosetta2 (DE3) and then purified after disruption by sonication. Purification scheme is as follows: his-tag affinity column, a desalting column, cut-off of the tag by TEV protease [27], his-tag affinity column again to remove the tag, and a size-exclusion column. All columns were purchased from GE Healthcare Bioscience, USA: a HisPrep column for his-tag affinity chromatography, a HiTrap Desalting column for desalting, and a Superdex-200 column for size-exclusion chromatography. The purified fusion protein was concentrated to 20.7 mg/ml for crystallization. Peptide samples for Ufd1 SHP box were synthesized by Peptron, Korea.

### Crystallization and structure determination

Crystallization conditions were initially searched using commercial screening kits, and the finally refined composition of well solution was 21%(w/v) PEG 8000, 2.9mM n-nonyl-β-thiomaltoside, 0.1 M HEPES, pH 7.5. The crystal was cryo-protected by quick soaking in a well solution containing additional 10%(v/v) glycerol. X-ray diffraction data were collected at the beamline 5C of Pohang Light Source, Korea. The raw diffraction images were processed, merged and scaled with *MOSFLM* and *SCALA* of *CCP4* program suite [28]. Molecular replacement calculation was carried out by *Phaser* [29], and the subsequent model rebuilding and refinement were performed iteratively using Coot and Refmac [30,31]. The coordinates and structure factors have been deposited in the Protein Data Bank with the accession number 5B6C (http://www.pdb.org).

### Isothermal titration calorimetry

Isothermal titration calorimetry (ITC) experiments were carried out using a ITC200 instrument (MicroCal Inc., USA). Titrations were carried out by injecting consecutive aliquots of p97 N domain (2.0 mM) into the ITC cell containing Ufd1 SHP peptide (0.050 mM) at 25°C. Binding stoichiometry, enthalpy, entropy, and binding constants were determined by fitting the data to a one-site binding model. The ITC data were fit using Origin 7.0 (MicroCal Inc., USA).

### Reagents and antibodies

DMEM, cycloheximide, anti-FLAG M2 affinity gels, and mouse monoclonal antibodies (mAbs) to α-tubulin and FLAG were purchased from Sigma-Aldrich. FBS and penicillin/streptomycin were obtained from Hyclone (Logan, UT). A mouse mAb to HA (16B12) and a goat polyclonal antibody to β-actin were obtained from Covance (Richmond, CA) and Santa Cruz Biotechnology, respectively. Mouse (9B11) and rabbit (71D10) mAbs to Myc were purchased from Cell Signaling Technology. Lipofectamine 2000 and Opti-MEM I were purchased from Thermo Fisher Scientific.

### Cell culture and transfection

HeLa cells were grown in DMEM supplemented with 10% FBS and penicillin/streptomycin at 37°C in a humidified atmosphere of 5% CO_2_ and 95% air. Cells were grown up to 70~80% confluence for transient transfection. For the Ufd1-p97 binding experiments, FLAG-Ufd1 and HA-p97 expression constructs (total 6 or 20 μg at a 1:1 ratio) or their relevant empty vectors as indicated conditions were mixed with Lipofectamine 2000 in Opti-MEM I, and the mixtures were added to cells for 1 day.

### Western blotting and immunoprecipitation

Cells were harvested in cold lysis buffer (50 mM Tris pH 7.4, 150 mM NaCl, 1 mM EDTA, 1 mM EGTA, 1 mM DTT, 1 mM Na_3_VO_4_, 5 mM NaF, and 1% Triton X-100) containing protease inhibitor cocktail tablets (Roche) and lysed with a TissueLyzer II (Qiagen) for 5 min. After clearance by centrifugation (15,000 × *g*, 20 min, 4°C), the protein concentration of the cell lysates was determined using bicinchoninic acid protein assay reagents (Pierce, Rockford, IL). For immunoprecipitation of FLAG-Ufd1 fusion proteins, cell lysates (0.5 mg) were incubated with 20 μl anti-FLAG M2 affinity gel for 4 h at 4°C. In case of immunoprecipitation of HA-p97 fusion proteins, cell lysates (1.0 mg) were incubated with 5.0 μg of anti-HA antibody for 4 h at 4°C, and then the immune complexes were captured with 50 μl protein G Sepharose 4 Fast Flow beads (GE Healthcare Life Sciences) for an additional 2 h. All immunoprecipitated samples were washed with the cell lysis buffer five times. The starting cell lysates and immunoprecipitates in Laemmli sample buffer were separated by SDS-PAGE on 8–10% resolving gels and transferred to nitrocellulose membranes (Schleicher & Schuell Bioscience, Germany). Following blocking with 5% nonfat milk in TBS containing 0.1% Tween-20 (TBST), membrane blots were incubated with primary antibodies against FLAG, HA and α-tubulin for 2 h at room temperature, washed three times with TBST, and further incubated with HRP-conjugated secondary antibodies (Zymed Laboratories, San Francisco, CA). The resulting immune complexes were detected using SuperSignal West Pico chemiluminescent substrate (Pierce, Rockford, IL).

### ER-associated degradation

For the degradation of the tyrosinase C89R mutant (Tyr^C89R^), cells plated on 35 mm-dishes were transfected with Myc-Tyr^C89R^ together with FLAG-Ufd1 or HA-p97 expression constructs (total 3 μg at a 1:1 ratio) or their relevant empty vectors for 16 h and then cell lysates were prepared as described above. For cycloheximide chase assays, the cotransfected cells were left untreated or treated with 50 μg/ml cycloheximide for up to 2 h. The protein levels of Myc-Tyr^C89R^ and transfected proteins were analyzed by Western blotting with mouse mAbs to Myc, FLAG and HA. Band intensities of the immunoblots were measured using NIH ImageJ software (National Institutes of Health, Bethesda, MD).

## Results

### Structure determination of the complex between the p97 N domain and the Ufd1 SHP box

Attempts to reconstitute and crystallize the complex between the p97 N domain and the Ufd1 SHP box were not successful for any of the constructs generated. Thus, we fused the p97 N domain (21–196; hereafter referred to as p97N) to the Ufd1 SHP box (225–235; hereafter referred to as Ufd1SHP) in a single polypeptide chain by using a long flexible linker. We designed two different fusion constructs: p97N-(GGS)_4_-Ufd1SHP and Ufd1SHP-(GGS)_4_-p97N. Both constructs were expressed well in *E*. *coli*, and then purified. The first construct produced a well-diffracting crystal, thus allowing a data set to be collected to 1.55 Å resolution limit (Fig 1A and Table 1). The structure was determined by molecular replacement method with the previously reported p97 N domain structure (Protein Data Bank entry 3QQ7) [32] as a search model. After positioning one molecule of the p97 N domain in the asymmetric unit, a well-defined electron density for the bound Ufd1SHP was observed in the difference map. The final refined model for p97N-Ufd1SHP complex contains residues Asn21 to Glu192 of the p97 N domain and the full 11-residue-long SHP box of Ufd1 (225-FRAFSGSGNRL-235). Four residues, Asp193 to Glu196, in the C-terminus of the p97 N domain, as well as twelve residues in the linker, (GGS)_4_, were not visible in the electron density map, even at the final stage of structure refinement, thus implying that these regions are disordered in the crystal lattice. The statistics for data collection and structure refinement are summarized in Table 1.

**Table 1:** Data collection and refinement statistics.

Ufd1 SHP box bound to p97 N domain^a^	
**Data collection**	
Space group	*H*3
Cell dimensions	
*a*, *b*, *c* (Å)	107.27, 107.27, 44.04
(°)	90.00, 90.00, 120.00
Resolution (Å)	30.00–1.55 (1.63–1.55)^b^
*R*_sym_	0.056 (0.182)
*I* / σ*I*	7.6 (4.1)
Completeness (%)	97.0 (82.2)
Redundancy	10.7 (7.5)
**Refinement**	
Resolution (Å)	30.00–1.55
No. reflections	25,310
*R*_work_ / *R*_free_	0.158 / 0.200
No. atoms	1,740
Protein	1,466
Water	274
*B*-factors	
Protein	16.74
p97N	15.4
Ufd1SHP	37.7
Water	26.91
R.m.s. deviations	
Bond lengths (Å)	0.023
Bond angles (°)	2.235

^a^One crystal was used for data collection and refinement.

^b^Values in parentheses are for highest-resolution shell.

**Fig 2:**
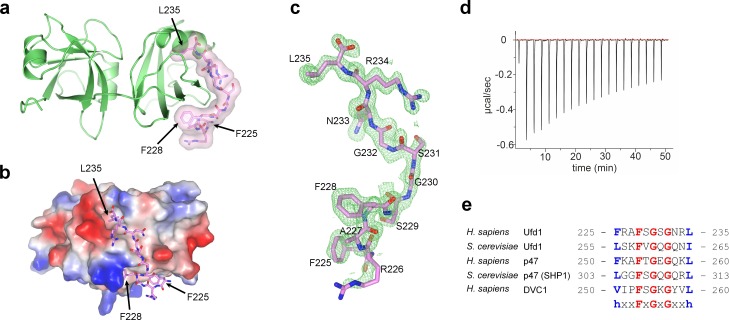
Features of Ufd1SHP binding to p97 N domain. **(a)** The p97 N domain is represented as a green ribbon as in Fig 1B, and the Ufd1 SHP box, pink, is illustrated as a stick model enclosed by a transparent surface model. The current view was obtained by an anti-clockwise 60° rotation from the view in Fig 1B around the in-plane horizontal axis. **(b)** The p97 N domain is illustrated as a surface model with electrostatic potential represented on the surface, and the Ufd1 SHP box is shown as a stick model in pink. The view was obtained by a clockwise 60° rotation from the view of Fig 2a around the in-plane vertical axis. **(c)** SIGMA-weighted *Fo-Fc* electron density map for the bound Ufd1SHP box contoured at 2.0 times the RMSD. **(d)** Isothermal titration calorimetry measurement of the interaction between the p97 N domain and the Ufd1 SHP box peptide. **(e)** Sequence alignment of the SHP boxes in yeast and human Ufd1, p47 and DVC1.

During the preparation of this manuscript, another research group reported a crystal structure for the complex between hexameric full-length p97 and the Ufd1 SHP box peptide at a moderate resolution of 3.08 Å, with less well-defined electron density for the SHP box, particularly for the central GSG region and the side chain orientations of the following C-terminal residues, as noted by authors [33]. In contrast, we used monomeric p97N rather than the full-length p97 to reconstitute the complex with Ufd1SHP. In addition, we constructed a fusion polypeptide containing both p97N and Ufd1SHP in a single chain to overcome their weak interaction and achieve higher occupancy of the bound Ufd1SHP in the crystal lattice. These two points may be the reasons why we were able to achieve such a high resolution of 1.55 Å, and obtain a clearly defined electron density map for this interaction (Fig 2C). The 1.55-Å resolution structure revealed the complete atomic details of this interaction, many of which have not previously been described in the recent report [33].

### High-resolution details of the interaction between the p97 N domain and the Ufd1 SHP box

As reported previously [2,3,32], the p97 N domain comprises two subdomains, Nn and Nc lobes; thus, it adopts an overall bilobed structure. The current crystal structure further reveals that the Ufd1SHP (225-FRAFSGSGNRL-235) binds only the Nc lobe, consistent with the recent report [33]. The SHP box wraps around the far-most face of the Nc lobe, with all eleven residues largely forming a random coil, except for a short stretch of β-strand from Asn233 to Arg234. This short β-strand, β^SHP^, binds to β11 at the outer edge of the central four-stranded β-sheet (β8-β9-β7-β11) of the Nc subdomain, thereby extending it to a five-stranded sheet (β8-β9-β7-β11-β^SHP^: four strands from p97N and one strand from Ufd1SHP) (Fig 1B). The entire Ufd1 SHP box adopts a bent conformation to establish elaborate interactions with the curved surface of the Nc lobe (Fig 2A and 2B). Two glycine residues (Gly230 and Gly232) generate a sharp kink in the middle of the SHP box, thereby enabling the bending of Ufd1SHP upon binding to p97N. These two glycine residues, which occupy the sixth and eighth positions in the eleven-residue SHP sequence, are strictly conserved through SHP-containing p97 adaptor proteins, reflecting their critical roles (Fig 2E).

**Fig 3:**
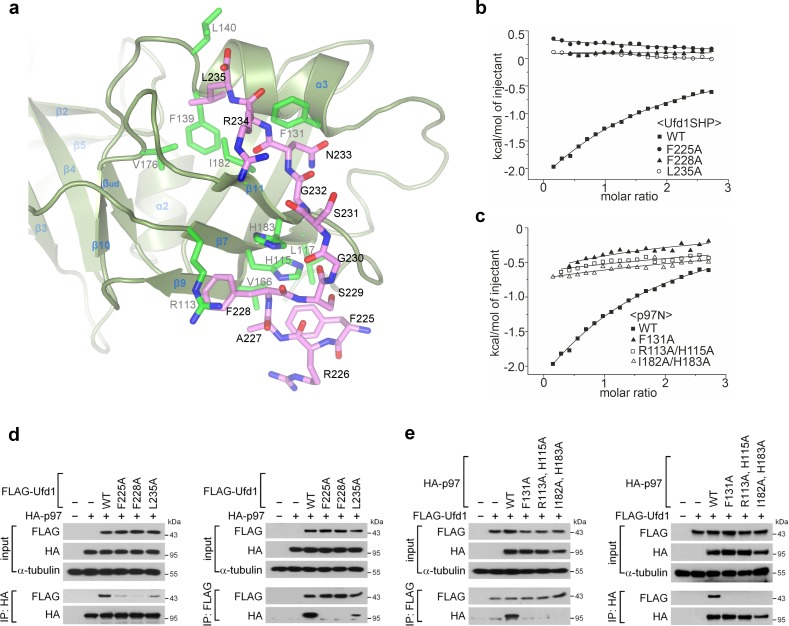
Structural details and mutational binding analyses of the interface. **(a)** Details of the binding interface are illustrated in stick models: the p97 N domain is shown in green, and the Ufd1 SHP box in pink. The view is similar to that in Fig 2B. **(b, c)** Isothermal titration calorimetry measurements of the interaction between the p97 N domain and the Ufd1 SHP box peptide: **(b)** p97N^WT^ with Ufd1SHP mutants, and **(c)** p97N mutants with Ufd1SHP^WT^. (**d, e**) Reciprocal coimmunoprecipitation. HeLa cells were cotransfected with expression plasmids encoding wild-type and mutant HA-p97 and FLAG-Ufd1 as indicated. At 24 h after transfection, the cell lysates were immunoprecipitated using anti-HA antibody and protein G beads or using anti-FLAG antibody-conjugated beads. The starting lysates (input) and immunoprecipitates (IP) were analyzed by Western blotting with anti-FLAG and anti-HA antibodies.α-tubulin was included as a loading control.

The Ufd1 SHP box binds to the p97 Nc subdomain primarily through hydrophobic interactions (Fig 2B), and the most prominent interactions are established by four residues of the Ufd1 SHP box (Phe225, Phe228, Asn233 and Leu235), whose side chains are positioned toward the p97 Nc subdomain (Figs 2B and 3A). Specifically, the phenyl ring of Phe225-Ufd1 sits on the hydrophobic patch formed by His115, Leu117 and Val166, forming a T-shaped π-π stack with the imidazole ring of His115. The phenyl ring of Phe228-Ufd1 stacks with the guanidinium group of Arg113, thereby establishing a cation-π interaction, and also forms a T-shaped π-π stack with the imidazole ring of His183. The side-chain amide plane of Asn233-Ufd1 stacks in parallel with the phenyl ring of Phe131, and the Leu235-Ufd1 at the C-terminal end inserts its side chain into the hydrophobic pocket constructed by Phe139, Leu140, V176, Ile182 and Phe131. Notably, the above three hydrophobic residues of Ufd1SHP (Phe225, Phe228 and Leu235) are highly conserved throughout SHP-containing p97 adaptor proteins (Ufd1, p47 and DVC1), which have been experimentally shown to bind to the p97 N domain (Fig 2E) [23,34,35].

In addition, several hydrogen bonds are also observed between main-chain atoms. First, the two main-chain oxygen atoms of Phe228-Ufd1 and Gly230-Ufd1 are weakly hydrogen-bonded to Nε2 of His183 at distances of 3.17 and 3.42 Å, respectively. Second, regarding to the short β-strand of Ufd1SHP (β^SHP^, Asn233 to Arg234) which binds to β11 of the Nc subdomain, the main-chain nitrogen and oxygen atoms of Asn233-Ufd1 (β^SHP^) are hydrogen-bonded to the main-chain oxygen and nitrogen atoms of Ile182 (β11 of p97N) in a typical way of bring β-strands together into a β-sheet. Notably, the Ufd1 SHP box contains two arginine residues, Arg226-Ufd1 and Arg234-Ufd1, whose side chains point in the opposite direction from the binding interface and therefore do not establish direct interactions with any p97 residues. This should be a reason why the side chains of these two arginine residues are not so well defined in the electron density as all the other residues of the Ufd1 SHP box (Figs 2C and 3A).

### Biochemical analysis of the interface

To biochemically verify the above structural observations, we performed mutational analyses for several key interface residues identified from the crystal structure. To begin with, we measured the binding affinity between the wild-types of the Ufd1 SHP box and the p97 N domain via isothermal titration calorimetry (ITC) by using a synthetic peptide of the Ufd1 SHP box and the recombinant p97 N domain (Fig 2D). The dissociation constant K_d_ was determined to be 221 ± 36 μM, implying that the interaction is quite weak. We then assessed whether mutations in the interface residues affect the binding affinity. Firstly, three mutant Ufd1SHP peptides, carrying F225A, F228A or L235A substitutions, were titrated against the wild-type p97 N domain. Next, three mutants of the p97 N domain, F131A, R113A/H115A and I182A/H183A, were tested with the wild-type Ufd1SHP peptide. Mutating the Ufd1SHP residues reduced the binding affinity to an undetectable level in the ITC experiment (Fig 3B), and mutating the p97N residues also abolished most of the binding affinity (Fig 3C).

p97-Ufd1 binding was also explored in cultured cells by reciprocal co-immunoprecipitation. To this end, we generated mammalian expression constructs of HA-tagged p97 and FLAG-tagged Ufd1 encoding full-length proteins containing the same series of mutations. After cotransfection into HeLa cells, p97 and Ufd1 in cell lysates and immunoprecipitation (IP) products were detected by Western blotting with anti-HA and anti-FLAG antibodies. First, wild-type or a mutant FLAG-Ufd1 was expressed together with wild-type HA-p97 (Fig 3D). The anti-HA IP products contained FLAG-Ufd1^WT^, as expected, but the three FLAG-Ufd1 mutants (F225A, F228A and L235A) co-precipitated with HA-p97 to a much lesser degree (Fig 3D, left). On the immunoprecipitation of the same cell lysates conversely by anti-FLAG antibody, the HA-p97 co-precipitated only weakly with FLAG-Ufd1^L235A^ and not at all with FLAG-Ufd1^F225A^ and FLAG-Ufd1^F228A^, in contrast to the strong co-precipitation with FLAG-Ufd1^WT^ (Fig 3D, right). The results from anti-HA and anti-FLAG IP experiments were in good agreement. Second, wild-type or mutant HA-p97 is co-expressed with wild-type FLAG-Ufd1 (Fig 3E). Both immunoprecipitation trials using anti-FLAG and anti-HA antibody commonly showed that all three p97 mutants (F131A, R113A/H115A and I182A/H183A) completely lost the binding affinity for FLAG-Ufd1^WT^.

### Functional implications in ER-associated degradation

**Fig 4:**
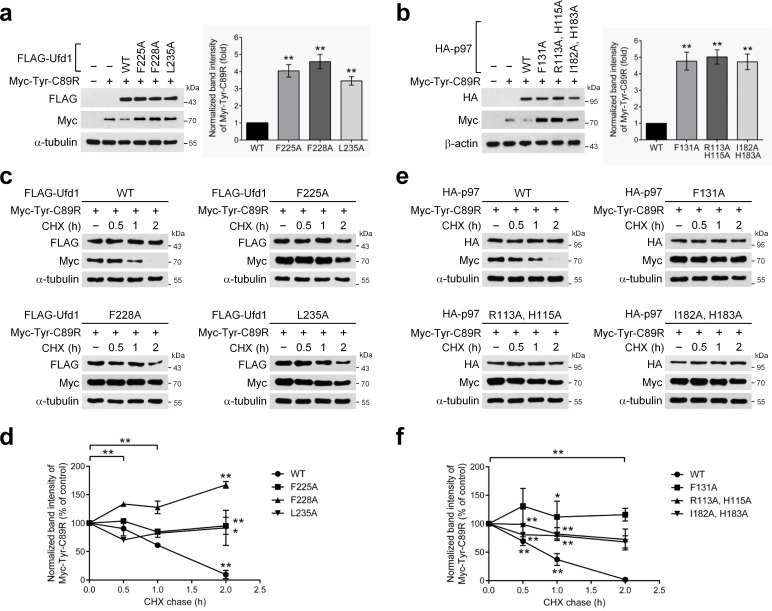
Effects of the binding-deficient mutants of p97 and Ufd1 on ERAD. The ERAD substrate Myc-Tyr^C89R^ was cotransfected with wild-type or mutant FLAG-Ufd1 **(a, c)** or HA-p97 **(b, e)** as indicated for 16 h. **(c, e)** Cells were further incubated with 50 μg/ml cycloheximide (CHX) for the indicated times. The levels of Myc-Tyr^C89R^ and the transfected proteins in cell lysates were analyzed by Western blotting with the indicated antibodies. α-tubulin or β-actin was included as a loading control. The protein levels of Myc-Tyr^C89R^ in the indicated mutant transfections were normalized to those of transfected Ufd1 **(a, c, d)** or p97 **(b, e, f)**, and quantified as fold-changes over such level of the corresponding WT transfection **(a, b)**, or quantified as percentage of the respective control (zero time) samples **(c‒f)**. Bar graphs in **(a, b)** represent mean ± SEM. **p<0.01. Line graphs in **(d, f)** represent mean ± S.D. **p<0.01, *p<0.05.

To investigate the functional significance of the structural details of the p97-Ufd1 interaction, we examined the effects of the above mutations on ER-associated degradation, a well-established cellular process involving both p97 and Ufd1. To this end, we assessed the protein level of Myc-tagged tyrosinase harboring the C89R mutation (tyrosinase-C89R or Tyr^C89R^), an ERAD substrate [36], with anti-Myc immunoblotting approximately 16 hours after cotransfection with wild-type or mutant HA-tagged p97 or FLAG-tagged Ufd1 (Fig 4A and 4B). The level of Myc-Tyr^C89R^ in the FLAG-Ufd1^WT^-cotransfected sample was lower than that observed with the expression of only Myc-Tyr^C89R^, indicating that FLAG-Ufd1^WT^ enhanced the degradation of Myc-Tyr^C89R^ (Fig 4A). In contrast, the protein level of Myc-Tyr^C89R^ was much higher in cells coexpressing the FLAG-Ufd1 mutant than in cells coexpressing FLAG-Ufd1^WT^: 4.05±0.37 fold for Ufd1^F225A^, 4.58±0.42 fold for Ufd1^F228A^ and 3.46±0.25 fold for Ufd1^L235A^. Similarly, the Myc-Tyr^C89R^ protein level was decreased after coexpression of HA-p97^WT^ but markedly increased after the HA-p97 mutants: 4.74±0.52 fold for p97^F131A^, 5.00±0.42 fold for p97^R113A/H115A^ and 4.70±0.46 fold for p97^I182A/H183A^ compared with HA-p97^WT^ (Fig 4B). Thus, all the tested Ufd1 and p97 interface mutants, compared with the wild types, showed substantially reduced tysoninase-C89R degradation. These results are consistent with the above binding affinity evaluation (Fig 3B–3E), in which all the same mutations greatly diminished the binding between Ufd1 and p97.

Given the well-established role for p97-Ufd1-Npl4 complex as a “segregase” that sequesters (or “segregates”) substrate proteins out of the ER membrane, thereby facilitating substrate translocation into the cytoplasm for subsequent proteasomal degradation [22], p97 and Ufd1 interface mutants can be reasonably assumed to delay protein degradation of tyrosinase-C89R, resulting in its accumulation. To verify this hypothesis, we performed standard cycloheximide (CHX) chase assays in the same experimental conditions as above and measured differences in the rate of degradation of Myc-Tyr^C89R^ between cells expressing wild-type and mutants of p97 or Ufd1 (Fig 4C–4F). As expected, Myc-Tyr^C89R^ underwent degradation CHX time-dependently upon Ufd1^WT^ expression (Fig 4C and 4D). In contrast, Ufd1^F225A^-, Ufd1^F228A^- and Ufd1^L235A^-expressing cells were much less active in degrading Myc-Tyr^C89R^, showing relatively prolonged retention time of the ERAD substrate (Fig 4C and 4D). Similarly, time-dependent decreases in Tyr^C89R^ protein levels were observed in the p97^WT^-expressing cells treated with CHX (Fig 4E and 4F). In a sharp contrast, however, Tyr^C89R^ remained at relatively high levels in the p97^F131A^-, p97^R113A/H115A^- and p97^I182A/H183A^ -expressing cells (Fig 4E and 4F), which is very similar to the results obtained from Ufd1 mutants. These results imply significant defects in degradation of Tyr^C89R^, an ERAD substrate, without the binding of Ufd1 to p97.

## Discussion

### Distinctive feature of SHP box as a p97N-binding module

The capacity of p97 to engage in diverse cellular processes is mediated by interacting with a variety of adaptor proteins at its N-terminal domain and C-terminal tail. Adaptors that bind the C-terminal tail utilize PUB or PUL domains as p97C-binding modules [37–39]. Far more adaptors bind the N-terminal domain, and five different p97N-binding modules included in these adaptors have been identified to date: UBX, UBD (also called ULD or UBX-L), VIM, VBM and SHP box [9,10]. Four p97N-binding modules (UBX, UBD, VIM, and VBM) have all been shown to bind to the cleft between the two subdomains of p97N; thus, the binding regions on the p97N surface cannot avoid overlapping to some extent [24,32,40–42]. However, in the present study, the SHP box was observed to bind at the far-most side of the Nc lobe, which is distant from the inter-subdomain cleft where the other four p97N-binding modules bind, and this observation was well consistent with a recent study [33].

The SHP box has been identified as a common motif (initially called binding site 1, BS1) in two major p97 adaptors, p47 and Ufd1-Npl4 [23]. Since then, more proteins have been demonstrated to bind p97 via SHP boxes. DVC1 recruits p97 via an SHP box to sites of DNA damage to extract translesion synthesis polymerase η from the DNA repair complex [34,35]. Derlin-1, an inactive intramembrane protease of the rhomoboid family and a central component of p97-interacting ER membrane complex involved in ERAD [43,44], also binds p97 via a C-terminal SHP box [44]. ASPL/TUG also contains a SHP box to bind p97 [45] and has previously been implicated in the insulin-stimulated redistribution of the glucose transporter GLUT4 and in the assembly of the Golgi compartment [46,47]. In addition, BLAST searches using a SHP box sequence as a query return a variety of proteins (http://blast.ncbi.nlm.nih.gov; result not shown), some of which have previously been reported to interact with p97 without recognition of the SHP box, such as RanBP2 (E3 SUMO ligase) at the nuclear pore complex (a SHP box at residues 1968 to 1978: 1968-FKGFSGAGEKL-1978) [48]. Thus, the SHP box seems to be as widely used as the UBX domain, which defines a large protein family, as a general p97N-binding module [49,50]. From this perspective, our complete, atomic-detail description of the SHP-p97 interaction should lay the groundwork for future functional and biochemical studies of SHP-containing protein family members, many of which await characterization.

### Assembly of p97-Ufd1-Npl4 complex and its segregase function

Because the SHP box of Ufd1 and the UBD of Npl4 bind to different sites on the surface of the p97 N domain, it may be structurally possible for the two modules of an Ufd1-Npl4 heterodimer to occupy the same N domain among the six in a hexameric assembly of p97. However, it may be also possible for the two modules of Ufd1-Npl4 to bind alternatively to two different N domains in a hexameric p97, because only one Ufd1-Npl4 complex binds to the hexameric p97 [20]. To our knowledge, there is no experimental evidence exclusively supporting either scenario, and this issue might be further addressed through biochemical and/or structural analyses of the whole p97-Ufd1-Npl4 complex.

**Fig 5:**
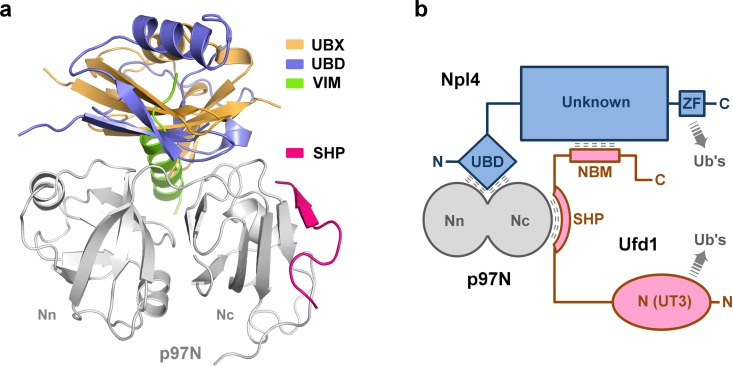
Distinct features of SHP as a p97N-binding module, and the assembly of p97-Ufd1-Npl4 complex. **(a)** Four p97N-binding modules in complex with the p97 N domain are illustrated in ribbon representations. The UBX of FAF1 (PDB, 3QC8) [41], the UBD of Npl4 (PDB, 2PJH) [24], the VIM of gp78 (PDB, 3TIW) [40], and the SHP of Ufd1 from the present study. **(b)** The p97-Ufd1-Npl4 complex is assembled by three different binary interactions. Ufd1 is colored in pink, Npl4 in blue, and p97 in gray. For simplicity, the binding of SHP (Ufd1) and UBD (Npl4) are pictured together on a single p97 N domain. However, whether two modules of the Ufd1-Npl4 complex bind to a single N domain or to two different N domains of a p97 hexamer remains unknown, as discussed in the text.

The assembly of p97-Ufd1-Npl4 complex is established through three different types of binary interactions between the three proteins (Figs 1A and 5): first, the Npl4 UBD binds to the inter-subdomain cleft of p97N; second, the Ufd1 SHP box binds to the far-most face of the Nc subdomain of p97N; and third, the Ufd1 NBM (named here as Npl4-binding motif) binds to the yet-uncharacterized central region of Npl4 [23]. The first and second interactions, Npl4UBD-p97N and Ufd1SHP-p97N, have been structurally characterized in detail through NMR and X-ray analyses, including the present study [24,33]. However, the Ufd1-Npl4 interaction has not been structurally or biochemically characterized. Previous work has revealed that the Npl4-binding motif resides between residues 258 and 275 in Ufd1, but the minimal sequence of the motif has yet to be identified [23].

The p97-Ufd1-Npl4 complex, organized by the above three binary interactions, reportedly binds to the ubiquitin chain of a client protein through the C-terminal zinc-finger motif of Npl4, the N domain of Ufd1, and the N domain of p97 [51–54]. The p97 exerts mechanical forces, which is generated from large conformational changes during ATP hydrolysis, on the ubiquitinated client protein via the Ufd1-Npl4 adaptor and thereby separates the client from its holding environment, such as a subcellular membrane or a multi-protein complex [6,22]. In the present study, we addressed one of the important structural issues for p97-Ufd1-Npl4 complex, particularly with respect to its assembly. The structural features of the interaction between the Ufd1 SHP box and the p97 N domain were clearly revealed in atomic details by determining the crystal structure at a high resolution of 1.55 Å. Furthermore, the observed structural details were corroborated by binding assays and functionally explored with respect to ER-associated degradation.
